# Two adjacent nuclear factor-binding domains activate expression from the human *PRNP *promoter

**DOI:** 10.1186/1756-0500-2-178

**Published:** 2009-09-09

**Authors:** Michael J Taheny, Nerik Izkhakov, Alexander A Vostrov, Wolfgang W Quitschke

**Affiliations:** 1 Department of Psychiatry and Behavioral Science, State University of New York at Stony Brook, Stony Brook, NY 11794-8101, USA

## Abstract

**Background:**

The transmissible spongiform encephalopathies (TSEs) comprise a group of fatal degenerative neurological diseases in humans and other mammals. After infection, the cellular prion protein isoform PrP^C ^is converted to the pathological PrP^SC ^scrapie isoform. The continued conversion of PrP^C ^to PrP^SC ^requires *de novo *endogenous PrP synthesis for disease progression. The human prion protein gene (*PRNP*) promoter was therefore investigated to identify regulatory elements that could serve as targets for therapeutic intervention.

**Findings:**

The human prion protein gene (*PRNP*) promoter from position -1593 to +134 relative to the putative transcriptional start site (+1) was analyzed by transient transfection in HeLa cells. Deletions from the 5' end between positions -1593 and -232 yielded little change in activity. A further 5' deletion at position -90 resulted in a decline in activity to a level of about 30% of the full-length value. DNase I footprinting of the region between positions -259 and +2 identified two adjacent protected domains designated as prpA (-116 to -143) and prpB (-147 to -186). Internal deletions combined with mobility shift electrophoresis and methylation interference assays indicated the presence of sequence specific nuclear factor complexes that bind to the prpA and prpB domains and activate expression from the human *PRNP *promoter in an additive fashion.

**Conclusion:**

Results from transient transfection, DNase I footprinting, mobility shift electrophoresis, and methylation interference experiments suggest that two DNase I protected domains designated as prpA and prpB are binding sites for as yet unidentified regulatory factors that independently activate expression from the *PRNP *promoter.

## Background

The TSEs comprise a group of fatal degenerative neurological diseases in mammals [1]. After infection, the endogenous cellular prion protein isoform PrP^C ^is converted to the pathological PrP^SC ^scrapie isoform. The continued conversion of PrP^C ^to PrP^SC ^requires de novo PrP synthesis [2-4]. These observations suggest that a reduction in PRNP expression could ameliorate the progression of TSEs.

The human *PRNP *contains two exons with the ORF located on exon 2 and the 5' untranslated region of the mRNA on exon 1 [5]. The *PRNP *is expressed at varying levels in many tissues and it is developmentally regulated [6,7]. *PRNP *expression was reported to be upregulated by copper ions, NGF, hypoxia, and hypoglycemia [8-12]. The *PRNP *promoter region has been characterized for the bovine, murine, rat, marsupial, and human genes [13-18]. It lacks a TATA box but has a high GC content and contains recognition sequences for numerous transcription factors [18]. Polymorphisms in the human and bovine promoters have been linked to increased susceptibility to prion diseases [19,20]. The human *PRNP *promoter was here further analyzed to identify additional regulatory mechanisms.

## Results and Discussion

### Transfection with PRNP promoter 5' deletions

**Figure 1:**
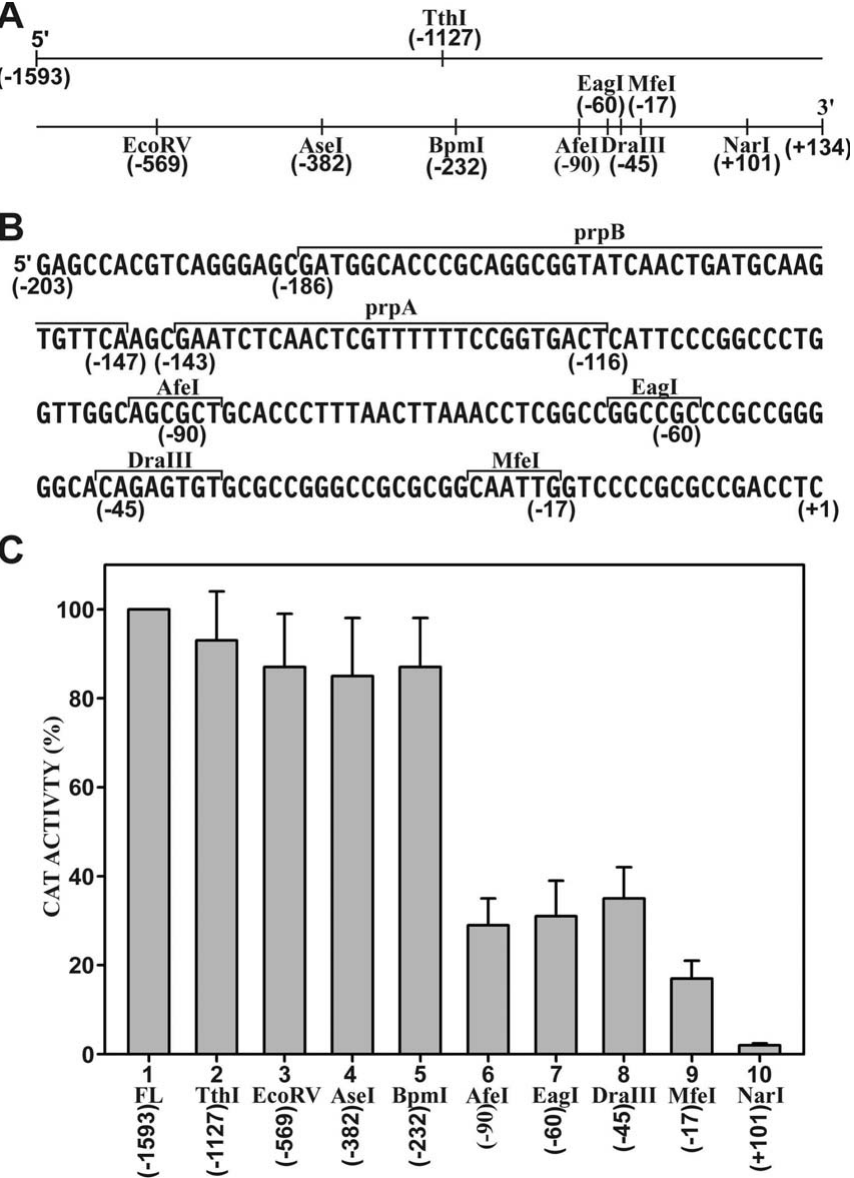
**Sequence of the *PRNP *promoter and expression analysis of 5' deletion constructs**. (A) Schematic representation of the *PRNP *promoter from position -1593 to +134 relative to the putative transcriptional start site (+1) [21]. The positions of relevant restriction sites used for deletion analysis are indicated. (B) Sequence of the proximal *PRNP *promoter from position -203 to +1. The positions of relevant restriction sites and nuclear factor binding domains prpA and prpB are delineated by brackets. (C) *PRPN *promoter activities in HeLa cells as determined by transient transfection. The full-length (FL) promoter construct (column 1) represents the sequence from position -1593 to +134 as indicated in A. The remaining 5' deletions (columns 2-10) were introduced at the restriction sites as shown in A and B. CAT activities were normalized to identical β-galactosidase activities. The activity of each deletion was expressed relative to the activity of the full-length promoter construct (100%). Each column shows the average value of 8-10 independent experiments with standard deviation.

The full-length *PRNP *promoter and its 5' deletions (Fig. 1A and 1B) were transiently transfected into HeLa cells. Expression from the promoter remained largely unchanged at full-length levels to position -232 (Bpm I) relative to the transcriptional start site (+1) [21](Fig. 1C). A further deletion at position -90 (Afe I) resulted in a significant decline in activity to about 30% of the full-length value (p < 0.0001). Additional statistically highly significant decreases in activity were observed at position -17 (Mfe I, p < 0.0001) and +101 (Nar I, p < 0.0001) [Fig. 1C]. These results indicated the presence of a significant activating domain between position -232 and -90. Additional domains that are essential for *PRNP *expression are likely to exist downstream from position -45. However, these were not further examined here since they are close to the transcriptional start site and presumably involve factors associated with transcriptional initiation.

Other similar transfection studies with *PRNP *promoter deletions have yielded alternative results [17,18]. In one report, variable promoter expression from deletions between positions -1543 and -284 was observed, suggesting the presence of positive and negative regulatory factors interacting with that region [17]. The reasons for these divergent observations may be related to differences in cell types, reporter systems, and constructs used.

### DNase I footprinting

**Figure 2:**
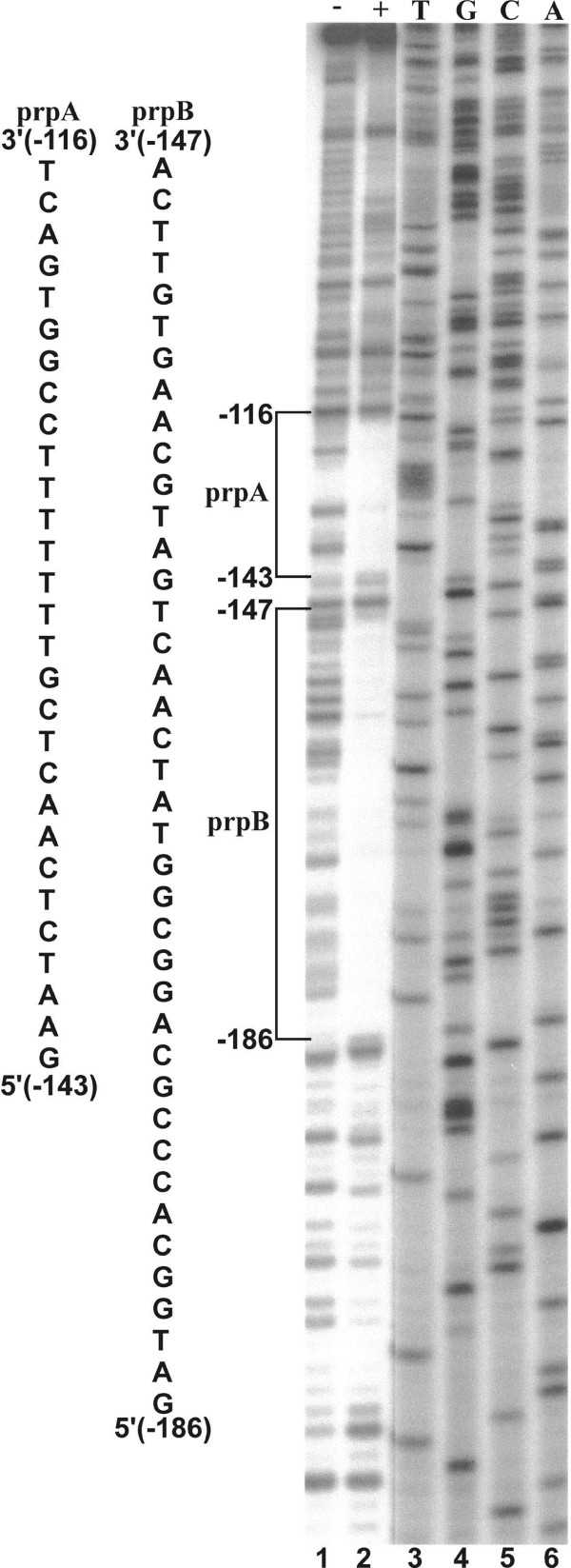
**DNase I footprinting of a *PRNP *promoter fragment extending from position -259 to +2. partial DNase I digestion was carried out either with (lane 2) or without (lane 1) HeLa cell nuclear extract**. A dideoxy sequencing reaction with the same primer was included for positional reference (lanes 3-6). Two prominent footprints separated by three base pairs were designated prpB and prpA (brackets). The sequence of the DNase I protected domains are shown in the left panel.

To determine if the region between positions -232 and -90 contains nuclear factor binding sites, a fragment extending from position -259 to +2 was generated by PCR and 5' end-labeled on the coding strand. The fragment was incubated with HeLa cell nuclear extract and partially digested with DNase I. Two prominent adjacent footprints designated as prpA (-116 to -143) and prpB (-147 and -186) were identified (Fig. 2).

### Mobility shift electrophoresis and methylation interference

**Figure 3:**
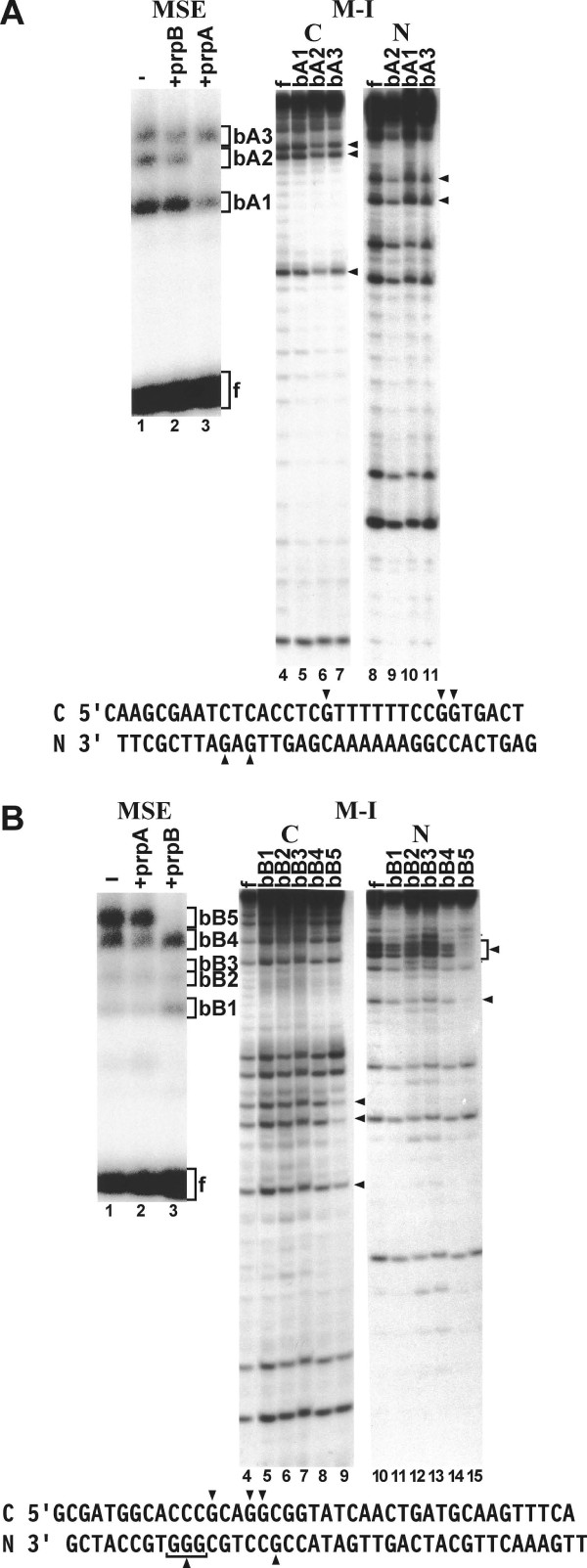
**Mobility shift electrophoresis (MSE) and methylation interference (M-I) with 5' end labeled double stranded oligonucleotides encompassing the sequences of DNase I protected domains prpA and prpB**. (A) MSE with 5' end labeled double stranded oligonucleotides encompassing the sequences of domain prpA (lane 1, and lower panel). Brackets show binding complexes (bA1-bA3) and the free oligonucleotide (f). Mobility shift competition was performed with a 20-fold molar excess of unlabeled oligonucleotide prpB (lane2) and prpA (lane 3). Methylated G residues within domain prpA that partially interfere with binding of complex bA2 on the coding [(C), lanes 4-7] and noncoding [(N), lanes 8-11] strands are indicated by arrowheads. Lanes 4 and 8 show the methylation patterns of the free oligonucleotides. Lower panel shows the sequence of the double stranded oligonucleotide used and G-residues that interfere with binding to complex bA2. (B) Same as in A except that the sequence of DNase I protected domain prpB was used. Mobility shift electrophoresis shows the formation of binding complexes bB1-bB5 with sequence-specific competition of complex bB5 (lanes 1-3). The same binding complex also exhibits binding interference with indicated methylated G residues (bracket, arrowheads) on coding (lanes 4-9) and noncoding (lanes 10-15) strands. Lanes 4 and 10 show the methylation patterns of the free oligonucleotides.

Mobility shift electrophoresis with double stranded oligonucleotide prpA resulted in three binding complexes designated as bA1-bA3 (Fig. 3A, lanes 1-3). To determine binding specificity, mobility shift competition was carried out with a 20-fold excess of unlabeled oligonucleotides prpB and prpA. No binding competition was detected with unlabeled oligonucleotide prpB. In contrast, self-competition with unlabeled prpA eliminated labeled complex bA2, and partially competed complex bA1. Complex bA3 remained unaffected. This suggested that the factors that bound to the prpA region were distinct from those that bound to the prpB region and that complex bA2 and possibly complex bA1 represented sequence specific binding. Methylation interference was carried out to identify DNA contact points and to confirm the specificity of the binding complexes. Partial interference with the formation of complex bA2 occurred with three methylated G residue on the coding strand (Fig 3A, lanes 4-7) and two G residues on the non-coding strand of oligonucleotide prpA (Fig 3A, lanes 8-10). Complexes bA1 and bA2 remained unaffected.

Mobility shift electrophoresis with labeled oligonucleotide prpB yielded five different binding complexes designated bB1-bB5. Of these complexes, bB1 and bB2 were exceedingly faint and complex bB3 was barely detectable. Mobility shift competition with excess unlabeled oligonucleotide prpA resulted in partial competition with complex bB4. The other complexes remained unaffected. Self-competition with oligonucleotide prpB completely competed labeled complex bB5 while the other complexes remained unaffected (Fig. 1B, lanes 1-3). This suggested that binding complex bB5 was sequence specific for the prpB region. Competition of prpA with complex bB4 indicated that this complex may share a binding motif common to both the prpA and prpB sequences. Partial methylation of domain prpB identified three G residues on the coding strand with partial (Fig. 3B, lanes 4-9) and four G residues on the non-coding strand (Fig 3B, lanes 10-15) with complete binding interference for complex bB5. All other binding complexes were unaffected by G-methylation.

The methylation interference experiments confirmed the results obtained by mobility shift electrophoresis. Binding complex bA2 on domain prpA and complex bB5 on prpB were sequence specific and they contained specific DNA contact points, defined by methylated G residues that interfered with complex formation to the respective domains.

In other related studies, the DNA binding sites for adjacent transcriptional factors YY1 and E4BP4 were characterized on the ovine *PRNP *promoter [22]. Those binding sites are conserved between mammalian species and they are present on the human *PRNP *promoter between positions -290 to -258. Consequently, they are located upstream from the prpA and prpB binding sites described here. *PRNP *expression was also found to be upregulated by SP1 and metal transcription factor-1 (MTF-1) [23]. However, the MTF-1 binding sites are located upstream (-594) and downstream (-90) from the prpA and prpB binding sites. Furthermore, while SP1 consensus sequences are numerous throughout the *PRNP *promoter, they do not overlap with the prpA or prpB motifs. Binding domain prpB also overlaps with a consensus sequence for the NF-IL6 (C/EBP) transcriptional regulator [18]. However, Dlaska and Weiss [24] have presented a DNase I footprint of NF-IL6 binding to the *iNOS *promoter and that footprint has a different configuration from the one observed here. In addition, the methylated G residues that interfere with binding to prpB do not overlap with the NF-IL6 consensus sequence. The 3' end of prpA also partially overlaps with a recognition sequence for transcription factor AP-1 [18]. However, binding of AP-1 to this domain is not supported by methylation interference results (Fig. 3). This suggests that the factors that bind to domains prpA and prpB are distinct from those previously described.

### Transfections with internal deletions of binding domains prpA and prpB

**Figure 4:**
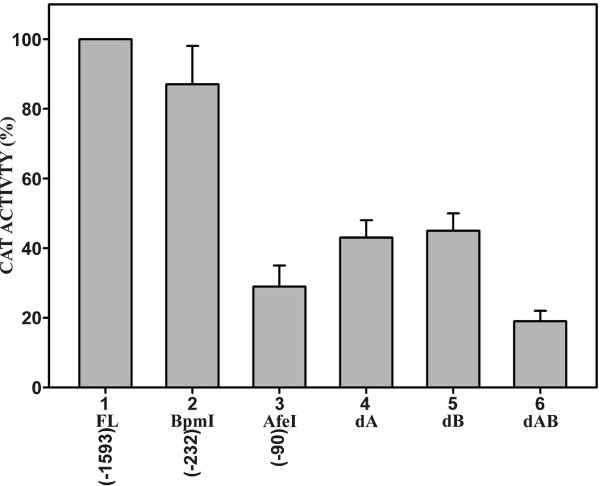
**Activity of internal deletions within the full-length *PRNP *(-1593 to +134) promoter construct (column 1) in HeLa cells**. Either binding domain prpA (column 4, dA) or prpB (column 5, dB), or both domains (column 6, dAB) were internally deleted. Deletions from the 5' end at restriction sites Bpm I (column 2, position -232) and Afe I (column 3, position -90) are shown for comparison. The activity of each deletion was expressed relative to the activity of the full-length promoter construct (100%). Each column shows the average of at least six independent experiments with standard deviation.

To further investigate the function of binding domains prpA and prpB, the two domains were internally deleted within a full-length *PRNP *promoter construct. Domains prpA and prpB were either deleted separately (Fig 4, dA and dB), or they were deleted together (Fig. 4, dAB). These internal deletions were then analyzed by transient transfection in HeLa cells. Individually deleting binding domain prpA or prpB, reduced expression from the *PRNP *promoter by approximately the same amount to about 43% of the wild-type value (Fig. 4, FL). However, deleting both domains together had an additive effect and expression was reduced to less than 20% of the wild type value. The activity from the 5' deletion terminating at position -90 (Fig. 4, Afe I) was about 30% of the full-length value. This construct is devoid of both binding domains prpA and prpB, but for currently unknown reasons its level of activity is intermediate between the constructs containing individual internal deletions of prpA and prpB (43%) and the combined deletion of both domains (18%) [Fig. 4]. It is conceivable that additional negative regulatory motifs exist that are removed in the 5' deletion at position -90. Overall, these results suggest that binding domain prpA and prpB act in an additive manner to activate *PRNP *promoter expression.

## Conclusion

A proximal promoter region extending from position -232 to +134 was identified that essentially confers full promoter activity in HeLa cells (Fig. 1). This region contains two nuclear factor-binding domains designated as prpA and prpB (Fig. 2). These domains are binding sites for distinct nuclear factors that are as yet unidentified (Fig. 3). The two domains independently activate expression from the *PRNP *promoter (Fig. 4). How these domains operate within context of the complete exon-intron structure of the endogenous *PRNP *gene or how they function within a neuronal cell background needs to be further investigated.

## Methods

### Cell cultures, transfections, and enzyme assays

HeLa cells (ATCC: CCL 2) were grown in DMEM supplemented with 5% fetal calf serum. Cells were transfected at 50% confluence with the ExGen 500 (Fermentas) transfection reagent according to the manufacturer's instructions. CAT and β-galactosidase assays were performed as described elsewhere [25]. CAT activities were quantitated with a BioRad GS-250 phosphor imager. The CAT activities resulting from the *APP *promoter constructs were normalized to corresponding β-galactosidase activities. Statistical significance (p-value) between selected final values was obtained with an unpaired t-test.

### Plasmids, promoter constructs, and oligonucleotides

A promoter region of the human *PRNP *was obtained from HeLa cell genomic DNA by PCR with primers complementary to the chromosome 20 genomic sequence from position 4,605,433 to 4,607,158 [NCBI: NT_011387.8]. The resulting promoter fragment extended from the 3' end of exon 1 (+134) to upstream position -1593 relative to a putative transcriptional start site (+1)[21]. Deletions from the 5'-end were generated by cutting with restriction enzymes at the indicated positions (Fig. 1A and 1B). All *PRNP *promoter constructs were analyzed in plasmid pCAT2bGAL [26]. This expression vector contains the bacterial genes *CAT *and *LacZ*. The *LacZ *gene is transcribed from the *β-actin *promoter and served as an internal control for experimental variations. The various *PRNP *promoter constructs were ligated into the mp18 polycloning site at the 5' end of the *CAT *gene. Internal deletions of the prpA and prpB domains were generated by *in vitro *mutagenesis [25,27]. Oligonucleotides were synthesized by Sigma Genosys or MWG Biotech and gel purified prior to use.

### Nuclear extracts and DNase I footprinting

Nuclear extracts were prepared from HeLa cells grown in suspension to a density of 5-8 × 10^5 ^cells/ml [25]. The final protein concentration in extracts was 10-15 mg/ml in buffer D containing 25 mM Hepes, pH 7.6, 100 mM KCl, 2 mM MgCl_2_, 0.2 mM EDTA, 1 mM DTT, 0.1 mM PMSF, and 10% glycerol. A promoter fragment extending from position -259 to +2 was amplified by PCR with a [^32^P] end-labeled forward primer complementary to the promoter sequence from position -259 to -235 and an unlabeled reverse primer complementary to the sequence from position -23 to +2. The resulting DNA fragment was purified by agarose gel electrophoresis in 0.5 × TBE. The labeled fragment (1 μl, 50 × 10^3 ^cpm) was preincubated with 8 μl of HeLa cell nuclear extract (+) or buffer D (-) in a total reaction volume of 33 μl as described below for mobility shift electrophoresis. After 30 min of incubation, the fragment was partially digested with 10-60 units of DNase I (Roche) for 10 min at 25°C. The reaction was stopped by adding 20 mM EDTA and 0.5% SDS, followed by phenol/chloroform extraction and ethanol precipitation. The dried pellet was solubilized in formamide loading buffer and electrophoresed in a 6% sequencing gel.

### Mobility shift electrophoresis and methylation interference

Double-stranded oligonucleotides comprising the sequences containing nuclear factor binding domains prpA or prpB (Fig. 3) were 5' end-labeled with [γ-^32^P]ATP [28]. Binding reactions were assembled with 5 μl of nuclear extract, 1 μl (50 × 10^3 ^cpm) of labeled oligonucleotide and 24 μl of buffer containing 40 mM Hepes pH 7.6, 2 mM MgCl_2_, 0.1 mM EDTA, 1 mM DTT, 50 mM KCl, 10% glycerol, supplemented with 2 μg of poly(dI-dC)-poly(dI-dC) [GE Healthcare]. After 30 min incubation at 25°C, the binding reaction products were separated by electrophoresis in 1% agarose gels containing 0.5 × TBE at 150 V constant voltage. The gels were dried and analyzed with a BioRad GS-250 Phosphor Imager. In competition assays, unlabeled oligonucleotides were premixed with labeled oligonucleotides at a 20-fold molar excess before adding them to the binding reaction.

Oligonucleotides comprising the sequences of binding domains prpA and prpB were partially methylated and 5' end-labeled on either the coding or non-coding strands as described [28]. After mobility shift electrophoresis, the binding complexes (Fig. 3) and the free oligonucleotide were isolated, cleaved with piperidine and separated on a 20% sequencing gel.

## List of abbreviations used

TBE: Tris-borate-EDTA; PMSF: phenylmethylsulfonyl fluoride; DMEM: Dulbecco's modified Eagle's medium; *PRNP: *prion protein gene; PrP: prion protein; ORF-open reading frame; TSE: transmissible spongiform encephalopathy; PCR: polymerase chain reaction; ATCC: American Type Culture Collection; CAT: chloramphenicol acetyltransferase; NGF: nerve growth factor.

## Competing interests

The authors declare that they have no competing interests.

## Authors' contributions

MT, WQ, and NI carried out molecular cloning and transfections. MT, NI, and AV performed DNase I footprinting and mobility shift electrophoresis. WQ and MT participated in methylation interference and *in vitro *mutagenesis experiments. All authors contributed to data analysis and WQ wrote the manuscript. All authors read and approved the final manuscript.
